# Comparison of three distinct clean air suits to decrease the bacterial load in the operating room: an observational study

**DOI:** 10.1186/s13037-015-0091-4

**Published:** 2016-01-07

**Authors:** Piotr Kasina, Ann Tammelin, Anne-Marie Blomfeldt, Bengt Ljungqvist, Berit Reinmüller, Carin Ottosson

**Affiliations:** Department of Clinical Science and Education, Orthopaedic Unit, Karolinska Institutet, Södersjukhuset, S-118 83 Stockholm, Sweden; Department of Medicine, Solna (MedS), Unit of Infectious Diseases, Karolinska Institutet, Stockholm, Sweden; Department of Energy and Environment, Division of Building Services Engineering, Chalmers University of Technology, Göteborg, Sweden

**Keywords:** Orthopaedic surgery, Surgical clothing, Ventilation, Operating room, Air-borne bacteria

## Abstract

**Background:**

Lowering air-borne bacteria counts in the operating room is essential in prevention of surgical site infections in orthopaedic joint replacement surgery. This is mainly achieved by decreasing bacteria counts through dilution, with appropriate ventilation and by limiting the bacteria carrying skin particles, predominantly shed by the personnel. The aim of this study was to investigate if a single use polypropylene clothing system or a reusable polyester clothing system could offer similar air quality in the operating room as a mobile laminar airflow device-assisted reusable cotton/polyester clothing system.

**Methods:**

Prospective observational study design, comparing the performance of three Clean Air Suits by measuring Colony Forming Units (CFU)/m^3^ of air during elective hip and knee arthroplasties, performed at a large university-affiliated hospital. The amount of CFU/m^3^ of air was measured during 37 operations of which 13 were performed with staff dressed in scrub suits made of a reusable mixed material (69 % cotton, 30 % polyester, 1 % carbon fibre) accompanied by two mobile laminar airflow units. During 24 procedures no mobile laminar airflow units were used, 13 with staff using a reusable olefin fabric clothing (woven polypropylene) and 11 with staff dressed in single-use suits (non-woven spunbonded polypropylene). Air from the operating field was sampled through a filter, by a Sartorius MD8, and bacterial colonies were counted after incubation. There were 6–8 measurements from each procedure, in total 244 measurements. Statistical analysis was performed by Mann–Whitney *U*-test.

**Results:**

The single-use polypropylene suit reduced the amount of CFU/m^3^ to a significantly lower level than both other clothing systems.

**Conclusion:**

Single-use polypropylene clothing systems can replace mobile laminar airflow unit-assisted reusable mixed material-clothing systems. Measurements in standardized laboratory settings can only serve as guidelines as environments in real operation settings present a much more difficult challenge.

## Background

Periprosthetic joint infection (PJI) is one of the most devastating complications after joint replacement surgery. In Sweden PJI is, together with loosening of the prosthesis, the most common reason for revision surgery after knee prosthesis surgery [1] and the second most common reason for revision after hip prosthesis surgery [2]. The consequences of these infections cause great physical and emotional suffering for the affected patients [3] and even increases mortality rate [4]. These complications create additional major costs for the health systems, in terms of vastly increased resource utilization through added and prolonged hospital stays and reoperations [5].

The necessity for limiting airborne bacteria in the operating room (OR), to establish a safer environment for orthopaedic implant surgery, has been well-established knowledge during the last decades [6, 7]. It is mainly achieved by a combination of adequate hygienic standards, good ventilation [8] and staff wearing clothing that reduces the emission of skin scales [9]. Requirements for clothing systems were set in 2009, as the European standard EN 13795 of the so-called Clean Air Suit [10].

Air quality can be assessed both by particle counting and microbiological sampling, but as the correlation between particle counts and microbial load is poor, it is preferable to measure Colony Forming Units (CFU) per cubic meter air [11].

In order to achieve as low CFU counts as possible, our department chose to complement our existing turbulent OR ventilation with additional mobile laminar airflow units (TOUL Meditech AB, Västerås, Sweden). These can lower the CFU-counts over the operating field and instrument table by creating laminar airflow through sterile filters [12].

Unfortunately these devices were perceived to influence the working environment in a negative way. TOUL-devices are placed above the instrument table and on stands, very close to the operating table, thus intruding on the area for the operating team. The overall complexity of the OR also limits the placement of these devices, making it difficult to ensure and verify their correct positioning. Additionally they also create a disturbing background noise.

The aim of our study was therefore to investigate if we could achieve equal air quality with a different clothing system, without the use of TOUL-devices.

## Methods

The study was conducted at the Dept. of Orthopaedics, Stockholm South General Hospital (Södersjukhuset AB), in 2013. The hospital holds 650 beds and employs 4400 co-workers, with Northern Europe’s largest emergency department. The orthopaedic department performs over 6000 operations per year.

The surgical procedures included in the study (elective arthroplasties of hips and knees) were all performed in standardized setting, in the same OR, equipped with turbulent ventilation, HEPA-filter and air-intake of 620 L/s. Experienced orthopaedic surgeons with one junior assistant performed the elective arthroplasty procedures of both hips and knees (cemented, uncemented and hybrid solutions). In total there were between 6 and 9 staff present at each operation, including both the operating personnel, as well as the passive observers responsible for data collection. We had strict door control with only one opening throughout all of the operations.

Three clothing systems were studied prospectively, all supplied as brand-new. There were 13 operations performed in our currently used reusable mixed material Mertex P-3477, Textilia AB (69 % cotton, 30 % polyester and 1 % carbon fibre) accompanied by two TOUL devices (joint airflow 220 L/min). In 13 operations, garments from a reusable olefin fabric, Olefin, Textilia AB (woven polypropylene/polyethene material) were used, without any TOUL device. The third clothing system, single-use BARRIER® Clean Air Suit, Mölnlycke Health Care (non-woven, spunbonded polypropylene), was used to perform 11 procedures, also without any TOUL device. Both the Mertex P-3477 dress and the BARRIER® Clean Air Suit, have cuffs at the bottom of the long legged pants and the short-sleeved shirts have cuffs at the arms, bottom and neckline. Olefin dresses had no cuffs at the bottom of the shirts and were therefore worn inside the pants.

The clothing systems were evenly distributed between morning and afternoon operations. During each operation, all staff present in the OR wore the same kind of clothes. These were later changed before next procedure.

Measurements were all performed by the same, experienced OR nurse, not involved in the surgical procedure. Air sampling was done with Sartorius Air Sampler MD8® (Sartorius AG, Goettingen, Germany). Air was sucked with standardized airflow of 100 L/min over a gelatine filter, placed 35–50 cm from the operating field, with special care regarding any splashes. It was changed every 10 min, with six to eight measurements per operation. Each filter was placed, immediately after removal, on a sterile blood agar plate and sent to the laboratory of Clinical Microbiology at the Karolinska University Hospital in Huddinge the same day. The plates were then incubated for 2 days at 35^O^C and the amount of colonies were then counted and expressed as CFU/m^3^.

Air sampling with condensate on the lid of the agar plate, macroscopic fluid or touch contamination, and damaged filters were excluded from analysis. Procedures with over 50 % excluded samples were subsequently dropped from the analysis.

When analysing the data, we calculated the median and mean values of CFU for each operation as well as each clothing system. The Olefin clothing and the BARRIER® Clean Air Suit were then independently compared to our existing TOUL-assisted Mertex P-3477 garment. As outliers and non-standard distribution was observed in all three clean air suits, the comparison was based on median values, using the Mann–Whitney *U*-test.

## Results

Our results are shown individually for each clothing system (Table 1, Table 2, and Table 3) and summarized for comparison (Table 4). The median values of the studied clothing systems were 20.0 CFU/m^3^ for the TOUL-assisted Mertex system, 22.5 CFU/m^3^ for the Olefin system and 12 CFU/m^3^ for the BARRIER® Clean Air Suit. We observed a wide spread of measurements between the different clothing systems, as well as between individual operations within the same clothing systems. The noticeably higher mean values (27.9 CFU/m^3^ of the TOUL-assisted Mertex system, 38.8 CFU/m^3^ of the Olefin clothing and 22.8 CFU/m^3^ of the BARRIER® Clean Air Suit) reflect the impact of the outliers, present in every clothing system and shown under the max CFU/m^3^ values in the tables. The reason of the high outlying values could not be traced back or explained by a specific date of surgery, temporary failure of ventilation or sterile processing department, malfunctioning Clean Air Suit items, nor the surgical teams or specific individuals.

**Table 1:** Results from 13 operations performed with staff dressed in Mertex P-3477, Textilia AB with two TOUL devices

Operation	Nr of persons in OR	Operation	CFU/m^3^			Measurements/excluded (reason)
			Median	Mean	Min - Max	
1	6	Uncemented THR	6.0	6.5	3–12	6/0
2	6	Cemented THR	55.0	63.8	23–100	7/1 (fluid on filter)
3	6	Cemented THR	23.0	22.7	15–30	7/1 (fluid on filter)
4	6	Cemented THR	33.0	43.4	21–100	7/2 (1 condensate, 1 damaged filter)
5	6	Cemented THR	8.0	18.5	7–18	7/3 (condensate)
6	6	Cemented THR	15.0	22.2	8–55	6/0
7	6	Cemented THR	8.0	10.8	6–20	6/1 (condensate)
8	6	Hybrid THR	46.0	61.3	23–148	7/0
9	6	Cemented THR	42.0	40.9	17–58	7/0
10	6	Cemented THR	24.0	24.9	12–40	7/0
11	6	Cemented THR	16.0	16.3	6–24	7/1 (condensate)
12	6	Uncemented THR	8.5	10.2	6–17	7/1 (damaged filter)
13	6	Cemented THR	13.0	12.7	1–28	7/0

*OR* Operating Room, *CRU* Colony Forming Units, *THR* Total Hip Replacement

**Table 2:** Results from 13 operations performed with staff dressed in Olefin, Textilia AB

Operation	Nr of persons in OR	Operation	CFU/m^3^			Measurements/excluded (reason)
			Median	Mean	Min - Max	
1	6	Uncemented THR	36.5	37.0	20–57	6/0
2	6	Cemented THR	2.5	2.7	0–6	6/0
3	6	Cemented THR	112.0	177.0	44–188	6/0
4	6	Cemented THR	66.0	106.0	50–228	6/0
5	6	Cemented THR	17.5	20.7	1–40	6/0
6	6	Cemented THR	5.0	7.3	1–18	7/1 (fluid on filter)
7	8	Cemented THR	37.5	35.2	22–48	6/0
8	8	Hybrid THR	22.5	24.5	14–40	6/0
9	8	Cemented THR	7.0	8.0	2–16	8/2 (1 damaged filter, 1 tactile contamination)
10	9	Cemented THR	^a^	^a^	^a^(2–6)	7/4 (condensate)
11	8	Cemented THR	^a^	^a^	^a^(2)	7/6 (condensate)
12	6	Uncemented THR	^a^	^a^	^a^(4–6)	7/5 (condensate)
13	6	Cemented THR	22.5	25.0	10–45	7/3 (condensate)

*OR* Operating Room, *CRU* Colony Forming Units, *THR* Total Hip Replacement

^a^Excluded from analysis due to >1/2 excluded measurements of each operation

**Table 3:** Results from 13 operations performed with staff dressed in BARRIER® Clean Air Suit, Mölnlycke Health Care

Operation	Nr of persons in OR	Operation	CFU/m^3^			Measurements/excluded (reason)
			Median	Mean	Min - Max	
1	9	Uncemented THR	7.0	7.3	3–14	6/0
2	8	Cemented THR	3.0	3.0	0–8	7/1 (fluid on filter)
3	8	Cemented THR	6.0	7.8	3–18	7/2 (1 fluid on filter, 1 condensate)
4	8	Cemented THR	6.5	7.8	2–16	6/0
5^a^	8	Cemented THR	5.0	6.7	3–13	7/1 (fluid on filter)
6	8	Cemented TKR	26.0	29.3	17–45	7/1 (fluid on filter)
7	8	Cemented TKR	28.5	30.0	11–50	6/0
8	8	Hybrid THR	22.5	20.0	5–35	6/0
9	8	Cemented THR	23.5	80.5	1–280	6/0
10	7	Cemented THR	47.5	46.3	25–71	7/1 (damaged filter)
11	8	Cemented THR	10.0	10.0	13–19	6/0

*OR* Operating Room, *CRU* Colony Forming Units, *THR* Total Hip Replacement, *TKR* Total Knee Replacement

^a^1 door opening

**Table 4:** Comparison of all tree clothing systems

Clothing system	CFU/m^3^			*p*-value^a^
	Median	Mean	Min - Max	
Mertex P-3477 + two TOUL devices	20.0	27.9	1–148	-
Olefin clothing	22.5	38.8	0–228	0.622
BARRIER® Clean Air Suit	12.0	22.8	0–280	0.009

*CFU* Colony Forming Units

^a^As compared to TOUL-assisted Mertex P-3477 clothing

Out of the total 244 measurements, we had to exclude 37 (15 %). This was done due to condensate on the lid of the agar plate (25 measurements), macroscopic fluid splash contaminations (7 measurements), damaged filter (4 measurements) and one tactile contamination. In three operations with the Olefin clothing, more than half of the measurements were excluded. Therefore, these three procedures were not regarded as representable and were consequently eliminated from the analysis. Conclusively we analyzed 201 measurement, 78 of which with the TOUL-assisted Mertex system, 58 with the Olefin clothing and 65 measurements with the BARRIER® Clean Air Suit.

The only door opening, on the fifth operation with BARRIER® Clean Air Suit, did not show any evident rise in measured CFU-values.

Our main finding was the lowest median CFU/m^3^ of the BARRIER® Clean Air Suit, which was significantly lower (p 0.009) and therefore superior to our standard clothing, the TOUL-assisted Mertex. The Olefin suit had the highest median and mean values of all three tested systems and did not show any significant difference to the TOUL-assisted Mertex system.

## Discussion

The most common OR environment in Sweden, for joint replacement surgery, is turbulent, mixed airflow combined with usage of reusable mixed (cotton/polyester) clothing. Previous studies have shown that polyester clothing is superior to both cotton and reusable mixed clothing (cotton/polyester). As a result of previous measurements [13] and evaluation of our working environment, our department used mixed garments with additional TOUL-devices, shown to further improve air quality [12].

Single-use polypropylene clothing has also been shown to be superior to reusable mixed clothing [14]. Our study shows that the single-use polypropylene clothing is able to reduce the bacterial counts more than both TOUL-assisted reusable mixed material clothing and reusable olefin clothing.

Door openings and increased traffic flow have a negative impact on bacterial contamination in the air [15]. In spite of our strict control, one opening was necessary during the 37 studied operations. Although it did not translate into any raise of CFU counts, this would be the expected outcome in repetitive cases.

Improving the ventilation system by rebuilding or new installation is appealing. However it is not possible in the short term or without erection of new facilities. Moreover decreasing the CFU counts by dilution through increased airflow with existing ventilation system is not easy. Heat generated by medical equipment and personnel, together with their diverse activities, different quantities or numbers, at times diverse placements and not infrequent (at multidisciplinary centers) special constellations, creates inevitably highly variable environments. Computed air velocity models have shown powerful vortex patterns even in assumed predictable, standardized settings. Reversed flows can build up inlet jets, between the patient and medical personnel, carrying air from floor level into the critical, operating field [16]. These streams can be both strong and unpredictable. Simultaneously the reach of mobile laminar airflow units is limited and sensitive to positioning, especially in a busy OR with limited space around the patient.

It is important for our patients that operating personnel can perform in an anticipated environment in the OR. It is thus necessary for all staff to use a well-known and commonly agreed clean air suit.

Manufacturers have to test their clothing systems in closed chambers and standardized settings for certification purposes. Ventilation systems are also assessed, before their usage in real life is commenced. Our ethical commitment to best patient care, does not allow testing these factors, as well as many other features, in real treatment prior to certification. The BARRIER® Clean Air Suit was our most stable performer, although its results varied noticeably. The variation, as the previously mentioned outliers of all three garments, could not be explained by variations in number of staff in the OR, the individuals within each operating team, the specific procedure performed, door openings or the ventilation. This difficulty reflects the complex and multifactorial relationship of real conditions of the ORs environment. Industrial standards therefore fail to predict the real outcome [14].

According to recommendations for air in operating rooms for infection-prone clean surgery the CFU-counts should be ≤5 CFU/m3 when clean air suits are worn [17]. The present study shows that this is not possible to achieve in our ORs with the present ventilation but also shows the importance of comparing available clothing systems to make the best choice. It is out of the scope of this study to make a cost benefit analysis on infection rates for the three clothing systems studied. With current prices the cost for the orthopaedic department, however, is the same regardless of which one of the clothing system is chosen.

Our department now continues clinical work with the BARRIER® Clean Air Suit. A new operating department, with better ventilation, is currently under construction and is expected to be ready and fully operational in 3 years. Concurrently we are looking at the market for additional devices to be placed inside the existing ORs, to further increase the turbulent ventilation.

## Conclusions

A single use polypropylene clothing system can offer better CFU reduction then both reusable olefin and mobile laminar airflow unit-assisted cotton/polyester clothing systems. Measurements in standardized settings can only serve as guidelines, as values in real operation settings can differ and be difficult to reproduce. Better theoretical and real evaluation models are required for satisfactory measurements and quality control for our patients.
